# Successful Control of Myasthenic Crisis After the Introduction of Ravulizumab in Myasthenia Gravis: A Case Report

**DOI:** 10.7759/cureus.74117

**Published:** 2024-11-20

**Authors:** Takafumi Uchi, Shingo Konno, Hideo Kihara, Hideki Sugimoto

**Affiliations:** 1 Neurology, Toho University Ohashi Medical Center, Tokyo, JPN

**Keywords:** case study, integrated treatment approach, myasthenia gravis, myasthenic crisis, ravulizumab

## Abstract

This case study describes the successful use of ravulizumab in treating a 71-year-old woman with myasthenia gravis experiencing a myasthenic crisis. The patient initially presented with hypernasality and dysphagia; her medical history included untreated, complicated type 1 diabetes. The patient received several treatments approved in Japan for general myasthenia gravis, including immunoadsorption plasmapheresis, tacrolimus, intravenous immunoglobulin, and intravenous methylprednisolone. Despite these treatments, the patient's condition fluctuated, and she ultimately experienced a myasthenic crisis, which required ventilator management. The introduction of ravulizumab, a complement inhibitor targeting the complement protein C5, marked a significant turning point in the patient's treatment. Ravulizumab improved the patient's respiratory function, allowing ventilator weaning and discharge from the hospital. To the best of our knowledge, this case is the first report of successful weaning from mechanical ventilation after treatment with ravulizumab in a patient with a myasthenic crisis. This finding suggests the efficacy of ravulizumab in the management of refractory myasthenia gravis and highlights the potential of novel therapeutic approaches and combination strategies in improving the condition. Future clinical trials are needed to assess the efficacy and safety of ravulizumab in larger, more diverse populations of patients with myasthenia gravis.

## Introduction

Myasthenia gravis (MG) is an autoimmune disorder characterized by impaired communication between nerves and muscles, leading to significant muscle weakness and fatigue. The core pathogenesis of MG lies in the disruption of the normal function of acetylcholine receptors (AChRs) at the neuromuscular junction, where nerve cells connect with the muscles they control [1].

Symptoms of MG typically worsen with prolonged muscle use as the reduced number of functional receptors becomes increasingly unable to meet the demands of continuous activity. Rest can temporarily alleviate symptoms by allowing some receptors to recover and resume normal function. MG is intimately connected to immune system dysregulation, as the production of autoantibodies plays a pivotal role in disease pathogenesis. In patients with MG, the immune system mistakenly targets components of the neuromuscular junction, leading to characteristic muscle weakness [2]. Corticosteroids, such as prednisolone, can help improve muscle strength by suppressing the immune system and reducing inflammation. Non-steroidal immunosuppressants approved and commonly used in Japan include cyclosporine and tacrolimus [3]; others not commonly used or not covered by health insurance in Japan include azathioprine and mycophenolate mofetil. These agents are used to dampen the immune response, thereby reducing the production of autoantibodies that interfere with neuromuscular transmission. For severe cases of MG, a combination of treatments may be used, including intravenous immunoglobulin therapy (IVIg) therapy, intravenous methylprednisolone (IVMP) pulse therapy, and plasmapheresis [4]. In patients with thymoma-associated MG or young patients with AChR-antibody (AChR-Ab)-positive MG, thymectomy has been shown to be effective [5]. These treatment options reflect the multifaceted approach to managing MG, aiming to improve the patient’s symptoms and quality of life.

Despite these advances, challenges remain for some patients. Myasthenic crisis - a complication of MG - is characterized as a severe exacerbation of MG symptoms, particularly muscle weakness [6]. It can escalate to respiratory failure, requiring urgent medical intervention. Management typically involves hospitalization, mechanical ventilation if necessary, and treatments such as IVIg therapy or plasmapheresis to quickly reduce AChR-Ab levels. Patients are considered to have difficult-to-treat MG when their disease becomes unresponsive to standard therapies. Difficult-to-treat MG can significantly affect the patient's quality of life, leading to physical limitations and emotional distress. These patients may need more aggressive or tailored treatments such as those described above. In particular, plasmapheresis is recommended for rapid symptom improvement in cases of acute exacerbation, including myasthenic crisis, to prevent post-thymectomy crisis or when there is insufficient symptom improvement after standard treatment. The procedure involves filtering the blood to remove pathogenic antibodies, specifically antibodies against AChR and muscle-specific tyrosine kinase from the plasma. By eliminating these antibodies, plasmapheresis can temporarily reduce the extent of the immune system's attack on the neuromuscular junction, leading to an improvement in muscle strength and function.

In cases where plasmapheresis cannot be fully administered because of an allergic reaction to blood product components, alternative treatments are necessary. One successful alternative treatment option is the use of additional immunosuppressive agents or immunomodulating therapies, depending on the patient's response and specific needs. Such agents may include corticosteroids, IVIg therapy, or monoclonal antibodies targeting different components of the immune system. Available biologics include eculizumab [7] and ravulizumab [8], which are complement inhibitors that target the complement protein C5. They prevent the cleavage of C5 into C5a and C5b, subsequently inhibiting the formation of the membrane attack complex (MAC) [8]. Efgartigimod is a neonatal fragment crystallizable receptor (FcRn) inhibitor that lowers the levels of pathogenic antibodies [9]. These agents help reduce the severe immune-mediated damage at the neuromuscular junction, which is a hallmark of myasthenic crisis.

Here, we report a case of MG in which the patient received a tailored treatment approach aimed at rapidly improving their condition by dampening the autoimmune activity at the neuromuscular junction and alleviating the symptoms of myasthenic crisis.

## Case presentation

**Figure 1:**
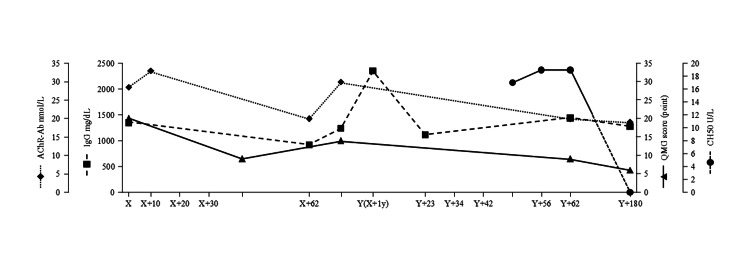
QMG scores, CH50 values, IgG levels, and AChR antibody levels over time X represents the date of first hospital admission, Y represents day X+1 year (365 days), i.e., the date of readmission exactly one year after the initial hospitalization AChR-Ab - acetylcholine receptor antibodies; CH50 - serum hemolytic complement activity; IgG - immunoglobulin G; QMG score - Quantitative Myasthenia Gravis score

A 71-year-old woman visited the Toho University Ohashi Medical Center complaining of difficulty swallowing. Two years prior to this visit, the patient experienced her first symptoms, which were hypernasality and dysphagia. Her medical history included complicated type 1 diabetes (glycated hemoglobin, 6.3%) for which she was not receiving medication. She had the following physical characteristics upon admission: height, 158 cm; weight, 44.5 kg; body mass index, 17.8 kg/m^2^. Notably, the patient had experienced unintentional weight loss of 5 kg over the past year. Assessment of her vital signs and general physical examination did not reveal any other abnormalities. On neurological examination, the patient exhibited ptosis and dysarthria but no facial muscle asymmetry. Her grip strength was measured at 10 kg for the right hand and 12 kg for the left hand. Her Quantitative Myasthenia Gravis (QMG) score, as defined by the Myasthenia Gravis Foundation of America [10], was 20 of 39 points on day X (date of first hospital admission; Figure 1).

**Figure 2:**
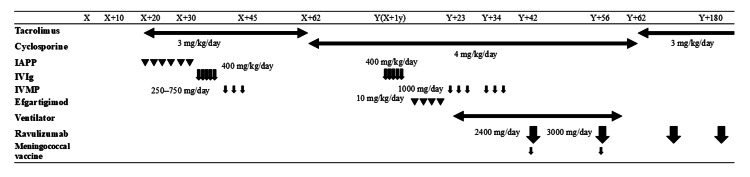
Schematic of the patient's treatment X represents the date of first hospital admission, Y represents day X+1 year (365 days), i.e., the date of readmission exactly one year after the initial hospitalization. Regarding the treatment choices after the second hospitalization (day Y), tacrolimus was changed to cyclosporine as a treatment for pancytopenia before hospitalization. Immunoadsorption plasmapheresis could not be used because of the occurrence of an allergic-like fever during the previous hospitalization. IVIg therapy was selected because it had been effective previously; however, it was not very effective during the second hospitalization. The use of a molecular-targeted drug was considered, and a neonatal Fc receptor inhibitor was introduced with the aim of reducing anti-acetylcholine receptor antibody levels. Unfortunately, the patient experienced a myasthenic crisis and was placed on a ventilator. In this situation, intravenous methylprednisolone therapy was administered without concern for initial exacerbation. Ravulizumab was initiated because further improvement was needed. Following ravulizumab initiation, the patient was able to be weaned off the ventilator, after which they were switched back to tacrolimus as there was no improvement in blood cell count, even with cyclosporine treatment, and the estimated glomerular filtration rate had decreased significantly. IAPP - immunoadsorption plasmapheresis; IVIg - intravenous immunoglobulin; IVMP - intravenous methylprednisolone pulse therapy

Repetitive nerve stimulation at 3 Hz showed waning on the median nerve. Based on these findings, the patient was diagnosed with generalized MG. Laboratory data revealed a normal blood count; her glycated hemoglobin was 6.2%, anti-glutamic acid decarboxylase-antibody level was 12.2 U/mL, and AChR-Ab level was 28.5 nmol/L (Figure 1). A timeline of the treatment she received is shown in Figure 2.

The initial treatment plan included immunoadsorption plasmapheresis and induction of tacrolimus at a dose of 3 mg/day from day X+20. However, this treatment was ineffective as the patient experienced fever, which may have been attributable to an allergic reaction to blood product components, specifically albumin. The treatment was changed to IVIg 400 mg/kg/day for five days from day X+20 to X+25. The patient experienced a slight improvement in dysphagia following IVIg treatment. Subsequently, IVMP was administered, starting at 250 mg/day and increasing to 750 mg/day at three-day intervals from day X+30 to day X+45. This treatment further improved the patient's dysphagia and allowed her to eat regular food. Her QMG score decreased from 20 to 9 points, and her AChR-Ab level decreased from 28.5 to 20 nmol/L on day X+62 (Figure 1) when she was discharged.

After hospital discharge, the patient's bloodwork showed a decrease in white blood cell count. The patient was switched from tacrolimus to cyclosporine, given the possibility that her decreased white blood cell count may have been tacrolimus-induced. She gradually experienced symptoms of dysphagia. She was re-admitted to the hospital on day Y (day X+1 year (365 days)) and received a second round of IVIg treatment, which was ineffective for improving her dysphagia. The patient's laboratory data on day Y are shown in Table 1.

**Table 1:** Patient's laboratory data on day Y (day X+1 year (365 days)) Day X is defined at the date of first hospital admission

Test	Measured value	Normal range
White blood cell count	2000/μL	3300–8600/μL
Red blood cell count	3.77 × 10^6^/μL	3.86–4.92 × 10^6^/μL
Hemoglobin	10.8 g/dL	11.6–14.8 g/dL
Platelet count	230 × 10^3^/μL	158–348 × 10^3^/μL
Total protein	6.5 g/dL	6.5–8.2 g/dL
Albumin	3.4 g/dL	3.5–5.0 g/dL
Total bilirubin	1.1 mg/dL	0.2–1.2 mg/dL
Aspartate aminotransferase	16 IU/L	12–33 IU/L
Alanine transaminase	8 IU/L	5–35 IU/L
Lactate dehydrogenase	330 IU/L	230–480 IU/L
Alkaline phosphatase	66 IU/L	38–150 IU/L
γ-glutamyl transferase	4 IU/L	13–64 IU/L
Blood urea nitrogen	13 mg/dL	8–20 mg/dL
Creatinine	0.4 mg/dL	0.6–1.2 mg/dL
Uric acid	3.2 mg/dL	4.0–7.0 mg/dL
Triglycerides	94 mg/dL	40–130 mg/dL
High-density lipoprotein cholesterol	81 mg/dL	35–70 mg/dL
Low-density lipoprotein cholesterol	108 mg/dL	65–139 mg/dL
Na	142 mmol/L	135–145 mmol/L
K	3.7 mmol/L	3.5–5.0 mmol/L
Cl	102 mmol/L	98–108 mmol/L
Ca	9.7 mg/dL	8.2–10.2 mg/dL
P	2.5 mg/dL	2.5–4.5 mg/dL
Mg	1.8 mg/dL	1.8–2.4 mg/dL
Creatine phosphokinase	46 IU/L	32–187 IU/L
C-reactive protein	3.4 mg/dL	0–0.3 mg/dL
Immunoglobin G	2356 mg/dL	700–1600 mg/dL
Hemoglobin A1C	6.2%	5.2–6.2%
Glucose	100 mg/dL	70–110 mg/dL

The patient's QMG score increased from 9 to 14 points, and her AChR-Ab levels increased from 20 to 30 nmol/L (Figure 1). Efgartigimod was then administered in four treatment cycles of once-weekly intravenous infusions (10 mg/kg) (four per cycle). However, after efgartigimod treatment initiation, the patient continued to experience dysphagia. This led to the development of aspiration pneumonia, and the patient experienced a myasthenic crisis, which necessitated ventilator management on day Y+23. The patient received two cycles of IVMP pulse therapy to wean off the mechanical ventilator. IVMP pulse therapy was selected instead of plasmapheresis to avoid a repeat allergic reaction to blood product components, given the reaction to the initial plasmapheresis. Despite IVMP pulse therapy, the patient's respiratory and swallowing functions did not improve enough to achieve ventilator weaning. Thus, on day Y+34, a tracheostomy was performed.

On day Y+42, ravulizumab 2400 mg/day was administered intravenously as the first dose of the treatment cycle, concurrent with a meningococcal vaccine. The vaccine was a necessary precaution given the increased risk of meningococcal infection with ravulizumab administration. Ravulizumab 3000 mg was given as the second treatment cycle 2 weeks later (day Y+56). The patient's serum hemolytic complement activity (CH50) levels were between 17.8 and 20.0 U/L after two cycles of ravulizumab treatment (Figure 1). Starting on day Y+58, the patient's respiratory function began to show notable improvement. The patient continued to gradually improve, which allowed for successful ventilator weaning, and she was taken off the ventilator on day Y+62. By day Y+74, her tracheostomy was successfully closed, and she was stable enough to be discharged from the hospital, marking a significant milestone in her recovery.

As the number of ravulizumab doses increased, the patient's complement activity levels changed markedly. The levels dropped below 12.0 U/L, or below the sensitivity of detection, following the third treatment cycle (Figure 1). There was a modest reduction in AChR-Ab levels from 30.0 nmol/L at day Y-10 to 20.5 nmol/L at day Y+62 (Figure 1). This decline indicated a positive response to combination treatment (IVMP treatment followed by ravulizumab), reflecting a decrease in the autoantibodies responsible for the symptomatic manifestations of MG. Post-discharge, the patient's condition was managed with tacrolimus 3 mg/day, and her white blood cell count remained stable. Additionally, ravulizumab 3000 mg was administered every two months as part of outpatient care.

## Discussion

This case study demonstrates a novel, innovative use of ravulizumab to manage a myasthenic crisis. The positive findings here provide compelling evidence of the therapeutic value of ravulizumab in the management of a difficult-to-treat case of myasthenic crisis. The role of ravulizumab in this patient's treatment deserves special attention, and the notable occurrence of myasthenic crisis during efgartigimod treatment cycles warrants closer examination. The initiation of efgartigimod treatment two weeks after IVIg therapy introduces a significant variable. IVIg therapy leads to an increased presence of immunoglobulin G (IgG) antibodies in the serum. Typically, a proportion of these antibodies, including potentially pathogenic AChR-Abs, enter the IgG recycling system. The FcRn-IgG interaction protects IgG from degradation and releases it back into circulation. However, the influx of IgGs due to IVIg treatment could saturate the IgG recycling system, inadvertently increasing the likelihood of pathogenic AChR-Abs being recycled back into the circulation, thus contributing to the persistence or exacerbation of myasthenic symptoms. By acting as a FcRn blocking agent, efgartigimod is designed to reduce the recycling of IgG antibodies [9], including pathogenic AChR-Abs. However, in the context of recent IVIg therapy, the overwhelming presence of IgG antibodies may diminish the efficacy of efgartigimod. This is because the FcRn receptors might already be occupied or overwhelmed by the antibodies introduced by IVIg therapy, leaving less capacity for efgartigimod to exert its therapeutic effect by blocking the recycling of pathogenic antibodies. In this case study, the short interval between IVIg therapy and efgartigimod treatment initiation likely contributed to the reduced effectiveness of efgartigimod in mitigating the patient's myasthenic crisis. This highlights the importance of considering the interactions between different treatments in the management of autoimmune conditions. The deliberate two-week interval between the administration of efgartigimod and ravulizumab was subsequently implemented to circumvent any potential adverse interactions that might arise from their concurrent use. Efgartigimod, by virtue of its mechanism of action of inhibiting globulin recycling, has the potential risk of negatively impacting the effectiveness of subsequent treatments such as ravulizumab. This could happen if the residual effects of efgartigimod interfere with the mechanism of action of ravulizumab, thereby diminishing its therapeutic effect. This approach underscores the importance of understanding the pharmacodynamics and pharmacokinetics of medications when using complex treatment regimens, ensuring that the sequencing and timing of drug administration are optimized to maximize therapeutic efficacy and patient outcomes. The patient did not receive prophylactic antibiotics, and the meningococcal vaccine was administered simultaneously with the first dose of ravulizumab, rather than adhering to the standard practice of administering the vaccine two weeks in advance [11]. The decision to administer the meningococcal vaccine simultaneously was based on a pragmatic approach to patient care. This approach takes into consideration the relatively low risk of community-acquired meningococcal infection within a hospital setting, where the patient is likely to be in a controlled environment with minimal exposure to infectious agents. Co-administration of the vaccine with ravulizumab allowed for a swifter initiation of treatment, which is potentially crucial in patients who need immediate therapeutic intervention.

The absence of a marked decrease in AChR-Ab levels following initial treatment with ravulizumab aligns with the expected pharmacological impact of this type of therapy on the complement system rather than a direct action on AChR-Abs. Although the CH50 value was not measured prior to ravulizumab administration, we assumed that it was in the normal range given that the patient did not have a history of infections that could be associated with low complement levels. The apparent delayed CH50 response following ravulizumab treatment, in contrast with the immediate reduction in CH50 values seen with eculizumab [12], aligns with the established understanding that CH50 values are variable with ravulizumab and do not serve as a reliable indicator of C5 inhibition [13]. Of note, ravulizumab is preferred as a maintenance therapy over eculizumab owing to its longer half-life, which allows for a longer dosing interval [14]. Given the previous finding of variable CH50 values with ravulizumab treatment [13] and the subtle change in CH50 values after the first ravulizumab administration, clinicians and researchers cannot rely solely on this value as a direct indicator of the immediate efficacy of the drug for MG. Notably, in this case, the CH50 value fell below the sensitivity of detection only after the third dose of ravulizumab. Because the CH50 value after the third dose was a better indicator of ravulizumab efficacy against MG than that after the first and second doses, the CH50 value cannot act as a reliable, objective indicator of ravulizumab efficacy in the early phase of treatment. Resistance to eculizumab or ravulizumab can also be determined by measuring genetic polymorphisms of the antibody binding site of C5 [15]. The therapeutic mechanism of ravulizumab, as with eculizumab, involves the immediate inhibition of the C5 component [16], effectively preventing the formation of the MAC at the neuromuscular junction. The inhibition of MAC formation is crucial in the treatment of MG, as it mitigates the complement-mediated damage that exacerbates muscle weakness. The improvement in muscle strength observed following ravulizumab administration may be attributed to the attenuation of MAC-induced damage at the neuromuscular junction, allowing for the recovery of AChR-Ab injured sites. The half-life of AChR at the normal mammalian neuromuscular junction ranges from six to 13 days [17]. Given the individual variability in response to complement inhibitors and the critical role of clinical symptoms in evaluating treatment efficacy, assessing the degree of improvement based on the patient's clinical presentation following ravulizumab administration is paramount. This approach ensures a comprehensive evaluation of treatment effectiveness, accounting for both the pharmacological action of the therapy and the unique physiological response of the patient. This case report describes a significant development in the therapeutic approach for MG, with a particular focus on managing crises. Ravulizumab may broaden the therapeutic horizon for patients with MG during acute episodes, with the potential for a role in the management of MG outside the hospital setting by contributing to the long-term stabilization of symptoms, suggesting a new direction for targeted intervention. Based on data from patients with atypical hemolytic uremic syndrome and paroxysmal nocturnal hemoglobinuria, ravulizumab demonstrates significant economic advantages compared with eculizumab [18,19], primarily because of its less frequent dosing schedule. These cost reductions, coupled with improvements in quality of life, suggest that ravulizumab could be a highly cost-effective and more accessible treatment option in the management of MG as well. Given the long dosing interval, sustained efficacy, and favorable safety profile, ravulizumab may be an attractive option for long-term outpatient maintenance therapy for AChR-Ab-positive generalized MG. The role of ravulizumab, particularly as an option for those unresponsive to traditional therapies, may be important in the changing treatment landscape for MG. The alignment of the effectiveness of ravulizumab with previous findings with eculizumab treatment [20-23] reinforces the utility of complement inhibitors in addressing severe or emergent MG symptoms.

## Conclusions

This case report represents the first report of successful ventilator weaning after ravulizumab treatment of a patient experiencing a myasthenic crisis. Our findings suggest the efficacy of ravulizumab in the management of difficult-to-treat MG and confirm CH50 as an unreliable objective measure of early ravulizumab efficacy. Instead, evaluations of efficacy should be based on clinical assessment of the individual patient.

The findings of this case report may contribute to the current understanding of novel therapeutic agents such as ravulizumab in the treatment of MG. Combining ravulizumab with other immunotherapeutic measures may offer a promising strategy, especially in patients for whom plasmapheresis is not an option. This integrated treatment approach demonstrates both the benefits and feasibility of developing more personalized, effective, and safer treatment protocols to improve the management of MG and potentially other complex autoimmune disorders. These findings illustrate the need for continuous exploration and validation of new treatment combinations and strategies to optimize outcomes in autoimmune diseases and highlight the potential of innovative treatment strategies in autoimmune diseases. However, future trials are crucial to assess the generalizability of the efficacy and safety of ravulizumab in larger, more diverse populations of patients with MG. Such studies will be essential in establishing the broader applicability and potential long-term benefits of ravulizumab in MG treatment.
